# Methyl 2-{2-[(*E*)-(2-hy­droxy-3-meth­oxy­benzyl­idene)amino]­ethyl­amino}­cyclo­pentene-1-carbodithio­ate

**DOI:** 10.1107/S1600536811002972

**Published:** 2011-01-29

**Authors:** Saeid Menati, Ali Kakanejadi, Abbas Taeb, Giuseppe Bruno, Hadi Amiri Rudbari

**Affiliations:** a Department of Chemistry, Science and Research Branch, Islamic Azad University, Tehran, Iran; b Department of Chemistry, University of Lorestan, Lorestan, Iran; c Dipartimento di Chimica Inorganica, Vill. S. Agata, Salita Sperone 31, Universita di Messina, 98166 Messina, Italy

## Abstract

In the title Schiff base compound, C_17_H_22_N_2_O_2_S_2_, which adopts an *E* configuration with respect to the imine C=N double bond, the C=N and N—C bond distances are 1.2789 (16) and 1.4546 (16) Å, respectively. In the mol­ecule there are intra­molecular O—H⋯N and N—H⋯S hydrogen bonds, and the CH=N—C substituent is almost coplanar with the benzene ring [C—N—C—C torsion angle = −178.80 (11)°]. The crystal packing is stabilized by inter­molecular C—H⋯O and C—H⋯π inter­actions involving the aromatic ring.

## Related literature

For properties and applications of Schiff base compounds, see: Sabater *et al.* (1999 ▶); Di Bella & Fragala (2002 ▶); Lecren *et al.* (2007 ▶); Güngör & Gürkan (2010 ▶). For related structures, see: Pereira *et al.* (2008 ▶); Kumar *et al.* (1995 ▶); Asadi *et al.* (2009 ▶).
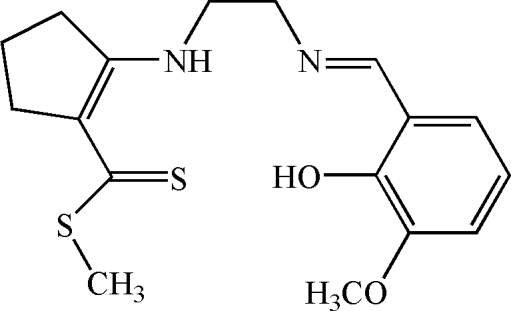


## Experimental

### 

#### Crystal data

C_17_H_22_N_2_O_2_S_2_
*M* *_r_* = 350.49
Triclinic, 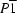

*a* = 7.7933 (2) Å
*b* = 10.3486 (2) Å
*c* = 11.9532 (3) Å
α = 108.038 (1)°
β = 93.349 (1)°
γ = 100.296 (1)°
*V* = 895.19 (4) Å^3^
*Z* = 2
Mo *K*α radiation
μ = 0.31 mm^−1^
*T* = 296 K
0.56 × 0.45 × 0.34 mm

#### Data collection

Bruker APEXII CCD diffractometer
Absorption correction: multi-scan (*SADABS*; Sheldrick, 1996 ▶) *T*_min_ = 0.678, *T*_max_ = 0.746
34525 measured reflections
4761 independent reflections
4235 reflections with *I* > 2σ(*I*)
*R*_int_ = 0.019

#### Refinement

*R*[*F* ^2^ > 2σ(*F* ^2^)] = 0.036
*wR*(*F* ^2^) = 0.109
*S* = 1.05
4761 reflections
211 parameters
H-atom parameters constrained
Δρ_max_ = 0.32 e Å^−3^
Δρ_min_ = −0.23 e Å^−3^

### 

Data collection: *APEX2* (Bruker, 2008 ▶); cell refinement: *SAINT* (Bruker, 2008 ▶); data reduction: *SAINT*; program(s) used to solve structure: *SHELXS97* (Sheldrick, 2008 ▶); program(s) used to refine structure: *SHELXL97* (Sheldrick, 2008 ▶); molecular graphics: *SHELXTL* (Sheldrick, 2008 ▶); software used to prepare material for publication: *SHELXTL*.

## Supplementary Material

Crystal structure: contains datablocks global, I. DOI: 10.1107/S1600536811002972/su2251sup1.cif

Structure factors: contains datablocks I. DOI: 10.1107/S1600536811002972/su2251Isup2.hkl

Additional supplementary materials:  crystallographic information; 3D view; checkCIF report

## Figures and Tables

**Table 1:** Hydrogen-bond geometry (Å, °) *Cg* is the centroid of the C11–C16 ring.

*D*—H⋯*A*	*D*—H	H⋯*A*	*D*⋯*A*	*D*—H⋯*A*
N1—H1⋯S2	0.86	2.32	3.0275 (11)	140
O2—H2⋯N2	0.82	1.85	2.5806 (14)	147
C9—H9*B*⋯O2^i^	0.97	2.51	3.1166 (16)	120
C1—H1*C*⋯*Cg*^ii^	0.96	2.95	3.617 (2)	128

Symmetry codes: (i) 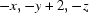; (ii) 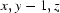.

**Fig. 1.**
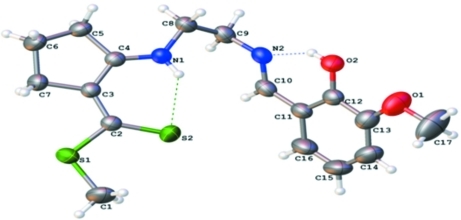
Molecular structure of the compound (1), with displacement ellipsoids drawn at the 50% probability level. The intramolecular N—H···S and O—H···N hydrogen bonds are shown as dashed lines.

**Fig. 2.**
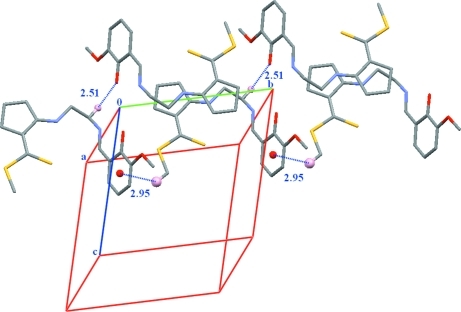
A view of the crystal packing of compound (1), with the C—H···O and the C–H···π interactions shown as dotted lines [see Table 1 for details; H-atoms not involved in these interactions have been omitted for clarity].

## supplementary crystallographic information

### Comment

Reflecting their usual relative ease of synthesis and excellent imine bonding properties, Schiff base compounds have been extensively investigated for more than a century. They have been employed in areas that include analytical and bioinorganic chemistry, non-linear optics, fluorescence studies, catalysis and materials chemistry (Sabater *et al.*, 1999; Di Bella *et al.*, 2002; Lecren *et al.*, 2007). The development of simple methods to produce asymmetric products remains an area of considerable research activity (Güngör *et al.*, 2010). In the other hand, it is well known that N and S atoms play a key role in the coordination of metals at the active sites of numerous metallobiomolecules. We are particularly interested in the synthesis and characterization of such asymmetric Schiff base compounds.

Three new asymmetric Schiff base compounds, (*E*)-methyl 2-(2-(2-hydroxy-3-methoxybenzylideneamino)ethylamino)cyclopent-1- enecarbodithioate (1), (*E*)-methyl 2-(2-(3,5-di-*tert-*butyl-2-hydroxybenzylideneamino)ethylamino) cyclopent-1-enecarbodithioate (2) and (*E*)-methyl 2-(2-(3-hydroxy-4-methoxybenzylideneamino)ethylamino) cyclopent-1- enecarbodithioate (3) have been prepared. Herein we report on the crystal structure of compound (1).

The molecular structure of compound (1) (Fig. 1) is similar to those of analogous derivatives (Pereira *et al.*, 2008; Kumar *et al.*, 1995; Asadi *et al.*, 2009). The title molecule adopts an E configuration with respect to the imine C═N double bond, with a C11—C10—N2—C9 torsion angle of -178.80 (11)°. The C12—O2 bond distance of 1.3377 (15) Å suggests that it is the phenol-imine tautomer. The contraction of the C10═N2 bond [1.2789 (16) Å] is also in agreement with the phenol-imine tautomer. As for the methoxy group, the O1—C13 and O1—C17 bond distances are 1.365 (2) and 1.420 (2) Å, respectively, and the C13—O1—C17 bond angle is 116.50 (17) Å. The planarity of the molecule is stabilized by intramolecular O—H···N and N–H···S hydrogen bonds (Fig. 1 and Table 1). However, there are no intermolecular hydrogen bonds associated with the methoxy group.

The crystal packing in compound (1) is stabilized by C—H···O and C—H···π interactions; the later involving the aromatic ring (C11—C16) and the C1—H1C H-atom (Fig. 2 and Table 1).

### Experimental

Methyl-2-{*N*-(2-aminoethane)}-amino-1-cyclopentenedithiocarboxylate (Hcden) was prepared by literature methods. The compounds (1), (2) and (3) were prepared by the addition of an equimolar amount of a methanolic solution of the appropriate benzaldehydr, 2-hydroxy-3-methoxybenzaldehyde, 3,5-di-*tert*-butyl-2-hydroxybenzaldehyde and 3-hydroxy-4-methoxybenzaldehyde, respectively, to a methanolic solution of Hcden. The products obtained were recrystallized from methanol/chloroform 1:1 (V:V).

### Refinement

The H-atoms were included in calculated positons and treated as riding atoms: O—H = 0.82 Å, N—H = 0.86 Å, C—H = 0.93, 0.96 and 0.97 Å for CH, CH_3_ and CH_2_ H-atons, respectively, with with *U*_iso_(H) = k × *U*_eq_(C), where k = 1.5 for OH and CH_3_ H-atoms, and k = 1.2 for all other H-atoms.

### Crystal data

**Table:**

C_17_H_22_N_2_O_2_S_2_	*Z* = 2
*M**_r_* = 350.49	*F*(000) = 372
Triclinic, *P*1	*D*_x_ = 1.300 Mg m^−^^3^
Hall symbol: -P 1	Mo *K*α radiation, λ = 0.71073 Å
*a* = 7.7933 (2) Å	Cell parameters from 9856 reflections
*b* = 10.3486 (2) Å	θ = 2.7–29.0°
*c* = 11.9532 (3) Å	µ = 0.31 mm^−^^1^
α = 108.038 (1)°	*T* = 296 K
β = 93.349 (1)°	Prismatic, black
γ = 100.296 (1)°	0.56 × 0.45 × 0.34 mm
*V* = 895.19 (4) Å^3^	

### Data collection

**Table:**

Bruker APEXII CCD diffractometer	4761 independent reflections
Radiation source: fine-focus sealed tube	4235 reflections with *I* > 2σ(*I*)
graphite	*R*_int_ = 0.019
φ and ω scans	θ_max_ = 29.1°, θ_min_ = 2.7°
Absorption correction: multi-scan (*SADABS*; Sheldrick, 1996)	*h* = −10→10
*T*_min_ = 0.678, *T*_max_ = 0.746	*k* = −14→14
34525 measured reflections	*l* = −16→16

### Refinement

**Table:**

Refinement on *F*^2^	Primary atom site location: structure-invariant direct methods
Least-squares matrix: full	Secondary atom site location: difference Fourier map
*R*[*F*^2^ > 2σ(*F*^2^)] = 0.036	Hydrogen site location: inferred from neighbouring sites
*wR*(*F*^2^) = 0.109	H-atom parameters constrained
*S* = 1.05	*w* = 1/[σ^2^(*F*_o_^2^) + (0.0602*P*)^2^ + 0.160*P*] where *P* = (*F*_o_^2^ + 2*F*_c_^2^)/3
4761 reflections	(Δ/σ)_max_ = 0.002
211 parameters	Δρ_max_ = 0.32 e Å^−^^3^
0 restraints	Δρ_min_ = −0.23 e Å^−^^3^

### Special details

**Table:**

Geometry. All esds (except the esd in the dihedral angle between two l.s. planes) are estimated using the full covariance matrix. The cell esds are taken into account individually in the estimation of esds in distances, angles and torsion angles; correlations between esds in cell parameters are only used when they are defined by crystal symmetry. An approximate (isotropic) treatment of cell esds is used for estimating esds involving l.s. planes.
Refinement. Refinement of *F*^2^ against ALL reflections. The weighted *R*-factor *wR* and goodness of fit *S* are based on *F*^2^, conventional *R*-factors *R* are based on *F*, with *F* set to zero for negative *F*^2^. The threshold expression of *F*^2^ > σ(*F*^2^) is used only for calculating *R*-factors(gt) *etc*. and is not relevant to the choice of reflections for refinement. *R*-factors based on *F*^2^ are statistically about twice as large as those based on *F*, and *R*- factors based on ALL data will be even larger.

### Fractional atomic coordinates and isotropic or equivalent isotropic displacement parameters (Å^2^)

**Table:**

	*x*	*y*	*z*	*U*_iso_*/*U*_eq_	
S1	0.34940 (5)	0.35488 (3)	0.20000 (3)	0.05017 (11)	
S2	0.29009 (5)	0.64159 (4)	0.22423 (3)	0.05473 (11)	
O1	−0.09386 (17)	1.17333 (16)	0.37397 (12)	0.0836 (4)	
O2	0.00507 (12)	1.04545 (10)	0.17106 (8)	0.0511 (2)	
H2	0.0495	1.0077	0.1123	0.077*	
N1	0.21120 (14)	0.62955 (11)	−0.03025 (9)	0.0435 (2)	
H1	0.2184	0.6727	0.0445	0.052*	
N2	0.23401 (13)	0.92834 (10)	0.05271 (10)	0.0433 (2)	
C1	0.3828 (3)	0.4423 (2)	0.35518 (14)	0.0785 (5)	
H1A	0.4744	0.5239	0.3731	0.118*	
H1B	0.4162	0.3817	0.3953	0.118*	
H1C	0.2761	0.4687	0.3810	0.118*	
C2	0.29524 (14)	0.47869 (11)	0.13773 (10)	0.0375 (2)	
C3	0.26114 (14)	0.42536 (11)	0.01462 (10)	0.0365 (2)	
C4	0.22487 (13)	0.49886 (11)	−0.06190 (9)	0.0360 (2)	
C5	0.20752 (18)	0.40864 (13)	−0.18882 (10)	0.0465 (3)	
H5A	0.3113	0.4324	−0.2252	0.056*	
H5B	0.1054	0.4178	−0.2339	0.056*	
C6	0.1871 (2)	0.26237 (15)	−0.18208 (13)	0.0625 (4)	
H6A	0.2465	0.2067	−0.2422	0.075*	
H6B	0.0639	0.2176	−0.1938	0.075*	
C7	0.2702 (2)	0.27904 (13)	−0.05846 (11)	0.0503 (3)	
H7A	0.2050	0.2117	−0.0272	0.060*	
H7B	0.3911	0.2676	−0.0596	0.060*	
C8	0.18503 (17)	0.70860 (13)	−0.10912 (11)	0.0461 (3)	
H8A	0.0618	0.7121	−0.1197	0.055*	
H8B	0.2189	0.6634	−0.1861	0.055*	
C9	0.29423 (16)	0.85464 (13)	−0.05752 (12)	0.0453 (3)	
H9A	0.4167	0.8509	−0.0428	0.054*	
H9B	0.2850	0.9041	−0.1138	0.054*	
C10	0.33243 (16)	0.95933 (13)	0.15062 (12)	0.0454 (3)	
H10	0.4421	0.9356	0.1493	0.054*	
C11	0.27848 (17)	1.03029 (12)	0.26352 (11)	0.0461 (3)	
C12	0.11586 (17)	1.06993 (12)	0.26856 (11)	0.0449 (3)	
C13	0.0665 (2)	1.13836 (16)	0.37903 (14)	0.0592 (3)	
C14	0.1797 (3)	1.16566 (19)	0.48081 (14)	0.0744 (5)	
H14	0.1466	1.2100	0.5541	0.089*	
C15	0.3412 (3)	1.1281 (2)	0.47537 (15)	0.0780 (5)	
H15	0.4165	1.1487	0.5447	0.094*	
C16	0.3908 (2)	1.06093 (17)	0.36880 (15)	0.0652 (4)	
H16	0.4993	1.0353	0.3658	0.078*	
C17	−0.1453 (3)	1.2484 (3)	0.4833 (2)	0.1217 (11)	
H17A	−0.1475	1.1949	0.5363	0.183*	
H17B	−0.2603	1.2659	0.4696	0.183*	
H17C	−0.0630	1.3351	0.5180	0.183*	

### Atomic displacement parameters (Å^2^)

**Table:**

	*U*^11^	*U*^22^	*U*^33^	*U*^12^	*U*^13^	*U*^23^
S1	0.0626 (2)	0.04592 (18)	0.04539 (17)	0.01308 (14)	0.00025 (14)	0.01981 (13)
S2	0.0818 (3)	0.04600 (18)	0.03766 (16)	0.02337 (16)	0.00973 (15)	0.00942 (13)
O1	0.0729 (7)	0.0936 (10)	0.0702 (8)	0.0283 (7)	0.0167 (6)	−0.0007 (7)
O2	0.0478 (5)	0.0523 (5)	0.0496 (5)	0.0163 (4)	−0.0034 (4)	0.0101 (4)
N1	0.0560 (6)	0.0407 (5)	0.0373 (5)	0.0170 (4)	0.0067 (4)	0.0138 (4)
N2	0.0452 (5)	0.0389 (5)	0.0488 (5)	0.0130 (4)	0.0043 (4)	0.0167 (4)
C1	0.1227 (16)	0.0735 (11)	0.0439 (7)	0.0214 (10)	0.0018 (9)	0.0266 (7)
C2	0.0363 (5)	0.0385 (5)	0.0389 (5)	0.0075 (4)	0.0053 (4)	0.0142 (4)
C3	0.0356 (5)	0.0345 (5)	0.0381 (5)	0.0071 (4)	0.0032 (4)	0.0105 (4)
C4	0.0321 (4)	0.0382 (5)	0.0369 (5)	0.0070 (4)	0.0033 (4)	0.0112 (4)
C5	0.0549 (7)	0.0438 (6)	0.0359 (5)	0.0074 (5)	−0.0013 (5)	0.0088 (5)
C6	0.0919 (11)	0.0417 (7)	0.0447 (7)	0.0121 (7)	−0.0077 (7)	0.0050 (5)
C7	0.0654 (8)	0.0371 (6)	0.0449 (6)	0.0130 (5)	−0.0009 (5)	0.0085 (5)
C8	0.0547 (6)	0.0463 (6)	0.0427 (6)	0.0181 (5)	0.0050 (5)	0.0183 (5)
C9	0.0466 (6)	0.0467 (6)	0.0522 (7)	0.0177 (5)	0.0123 (5)	0.0240 (5)
C10	0.0434 (6)	0.0392 (6)	0.0575 (7)	0.0114 (4)	0.0007 (5)	0.0208 (5)
C11	0.0531 (6)	0.0378 (5)	0.0486 (6)	0.0098 (5)	−0.0049 (5)	0.0174 (5)
C12	0.0517 (6)	0.0365 (5)	0.0459 (6)	0.0074 (5)	0.0000 (5)	0.0146 (5)
C13	0.0685 (9)	0.0524 (7)	0.0531 (8)	0.0121 (6)	0.0091 (6)	0.0119 (6)
C14	0.1066 (14)	0.0683 (10)	0.0437 (7)	0.0165 (10)	0.0050 (8)	0.0136 (7)
C15	0.1076 (14)	0.0729 (11)	0.0508 (8)	0.0220 (10)	−0.0196 (9)	0.0196 (8)
C16	0.0735 (9)	0.0595 (8)	0.0619 (9)	0.0192 (7)	−0.0176 (7)	0.0202 (7)
C17	0.0930 (15)	0.127 (2)	0.1020 (17)	0.0243 (15)	0.0333 (13)	−0.0292 (16)

### Geometric parameters (Å, °)

**Table:**

S1—C2	1.7666 (11)	C6—H6A	0.9700
S1—C1	1.7740 (16)	C6—H6B	0.9700
S2—C2	1.6918 (12)	C7—H7A	0.9700
O1—C13	1.365 (2)	C7—H7B	0.9700
O1—C17	1.420 (2)	C8—C9	1.5134 (18)
O2—C12	1.3377 (15)	C8—H8A	0.9700
O2—H2	0.8201	C8—H8B	0.9700
N1—C4	1.3126 (15)	C9—H9A	0.9700
N1—C8	1.4541 (15)	C9—H9B	0.9700
N1—H1	0.8595	C10—C11	1.4493 (19)
N2—C10	1.2789 (16)	C10—H10	0.9300
N2—C9	1.4546 (16)	C11—C12	1.3991 (18)
C1—H1A	0.9600	C11—C16	1.4055 (18)
C1—H1B	0.9600	C12—C13	1.4037 (19)
C1—H1C	0.9600	C13—C14	1.382 (2)
C2—C3	1.3926 (15)	C14—C15	1.381 (3)
C3—C4	1.4046 (15)	C14—H14	0.9300
C3—C7	1.5124 (16)	C15—C16	1.364 (3)
C4—C5	1.4984 (15)	C15—H15	0.9300
C5—C6	1.521 (2)	C16—H16	0.9300
C5—H5A	0.9700	C17—H17A	0.9600
C5—H5B	0.9700	C17—H17B	0.9600
C6—C7	1.5243 (19)	C17—H17C	0.9600
			
C2—S1—C1	104.65 (7)	N1—C8—C9	109.96 (10)
C13—O1—C17	116.50 (17)	N1—C8—H8A	109.7
C12—O2—H2	109.5	C9—C8—H8A	109.7
C4—N1—C8	126.44 (10)	N1—C8—H8B	109.7
C4—N1—H1	116.8	C9—C8—H8B	109.7
C8—N1—H1	116.7	H8A—C8—H8B	108.2
C10—N2—C9	119.45 (11)	N2—C9—C8	110.36 (10)
S1—C1—H1A	109.5	N2—C9—H9A	109.6
S1—C1—H1B	109.5	C8—C9—H9A	109.6
H1A—C1—H1B	109.5	N2—C9—H9B	109.6
S1—C1—H1C	109.5	C8—C9—H9B	109.6
H1A—C1—H1C	109.5	H9A—C9—H9B	108.1
H1B—C1—H1C	109.5	N2—C10—C11	122.07 (11)
C3—C2—S2	126.69 (9)	N2—C10—H10	119.0
C3—C2—S1	112.17 (8)	C11—C10—H10	119.0
S2—C2—S1	121.14 (7)	C12—C11—C16	119.61 (14)
C2—C3—C4	126.43 (10)	C12—C11—C10	120.49 (11)
C2—C3—C7	124.42 (10)	C16—C11—C10	119.90 (13)
C4—C3—C7	109.03 (10)	O2—C12—C11	122.13 (12)
N1—C4—C3	126.24 (10)	O2—C12—C13	118.47 (12)
N1—C4—C5	122.93 (10)	C11—C12—C13	119.40 (12)
C3—C4—C5	110.81 (10)	O1—C13—C14	125.93 (15)
C4—C5—C6	103.91 (10)	O1—C13—C12	114.69 (14)
C4—C5—H5A	111.0	C14—C13—C12	119.38 (15)
C6—C5—H5A	111.0	C15—C14—C13	121.06 (16)
C4—C5—H5B	111.0	C15—C14—H14	119.5
C6—C5—H5B	111.0	C13—C14—H14	119.5
H5A—C5—H5B	109.0	C16—C15—C14	120.29 (15)
C5—C6—C7	105.86 (10)	C16—C15—H15	119.9
C5—C6—H6A	110.6	C14—C15—H15	119.9
C7—C6—H6A	110.6	C15—C16—C11	120.26 (16)
C5—C6—H6B	110.6	C15—C16—H16	119.9
C7—C6—H6B	110.6	C11—C16—H16	119.9
H6A—C6—H6B	108.7	O1—C17—H17A	109.5
C3—C7—C6	104.22 (10)	O1—C17—H17B	109.5
C3—C7—H7A	110.9	H17A—C17—H17B	109.5
C6—C7—H7A	110.9	O1—C17—H17C	109.5
C3—C7—H7B	110.9	H17A—C17—H17C	109.5
C6—C7—H7B	110.9	H17B—C17—H17C	109.5
H7A—C7—H7B	108.9		
			
C1—S1—C2—C3	−178.88 (11)	N1—C8—C9—N2	−64.63 (13)
C1—S1—C2—S2	1.58 (11)	C9—N2—C10—C11	−178.80 (11)
S2—C2—C3—C4	3.59 (17)	N2—C10—C11—C12	−1.23 (19)
S1—C2—C3—C4	−175.92 (9)	N2—C10—C11—C16	179.42 (12)
S2—C2—C3—C7	179.08 (10)	C16—C11—C12—O2	178.90 (13)
S1—C2—C3—C7	−0.43 (15)	C10—C11—C12—O2	−0.45 (18)
C8—N1—C4—C3	175.70 (11)	C16—C11—C12—C13	−0.74 (19)
C8—N1—C4—C5	−2.59 (19)	C10—C11—C12—C13	179.91 (12)
C2—C3—C4—N1	−2.38 (19)	C17—O1—C13—C14	2.1 (3)
C7—C3—C4—N1	−178.45 (11)	C17—O1—C13—C12	−177.20 (19)
C2—C3—C4—C5	176.08 (11)	O2—C12—C13—O1	−0.1 (2)
C7—C3—C4—C5	0.01 (13)	C11—C12—C13—O1	179.54 (13)
N1—C4—C5—C6	−166.44 (12)	O2—C12—C13—C14	−179.49 (14)
C3—C4—C5—C6	15.04 (14)	C11—C12—C13—C14	0.2 (2)
C4—C5—C6—C7	−23.83 (16)	O1—C13—C14—C15	−178.55 (18)
C2—C3—C7—C6	168.79 (12)	C12—C13—C14—C15	0.7 (3)
C4—C3—C7—C6	−15.05 (15)	C13—C14—C15—C16	−1.1 (3)
C5—C6—C7—C3	23.93 (16)	C14—C15—C16—C11	0.5 (3)
C4—N1—C8—C9	−140.36 (12)	C12—C11—C16—C15	0.4 (2)
C10—N2—C9—C8	111.43 (12)	C10—C11—C16—C15	179.77 (15)

### Hydrogen-bond geometry (Å, °)

**Table:**

Cg is the centroid of the C11–C16 ring.

**Table:**

*D*—H···*A*	*D*—H	H···*A*	*D*···*A*	*D*—H···*A*
N1—H1···S2	0.86	2.32	3.0275 (11)	140
O2—H2···N2	0.82	1.85	2.5806 (14)	147
C9—H9B···O2^i^	0.97	2.51	3.1166 (16)	120
C1—H1C···Cg^ii^	0.96	2.95	3.617 (2)	128

Symmetry codes: (i) −*x*, −*y*+2, −*z*; (ii) *x*, *y*−1, *z*.
